# Tolerability and efficacy of the cancer vaccine UV1 in patients with recurrent or metastatic PD-L1 positive head and neck squamous cell carcinoma planned for first-line treatment with pembrolizumab – the randomized phase 2 FOCUS trial

**DOI:** 10.3389/fonc.2024.1283266

**Published:** 2024-02-07

**Authors:** Anna Brandt, Christoph Schultheiss, Konrad Klinghammer, Philippe Schafhausen, Chia-Jung Busch, Markus Blaurock, Axel Hinke, Mareike Tometten, Andreas Dietz, Urs Müller-Richter, Dennis Hahn, Jürgen Alt, Alexander Stein, Mascha Binder

**Affiliations:** ^1^ Division of Medical Oncology, University Hospital Basel, Basel, Switzerland; ^2^ Laboratory of Translational Immuno-Oncology, Department of Biomedicine, University and University Hospital Basel, Basel, Switzerland; ^3^ Department of Hematology and Oncology, Charité - Universitätsmedizin Berlin, Humboldt-Universität zu Berlin, and Berlin Institute of Health, Berlin, Germany; ^4^ Department of Oncology, Hematology and Bone Marrow Transplantation with Section of Pneumology, University Medical Center Hamburg-Eppendorf, Hamburg, Germany; ^5^ Department of Otorhinolaryngology, Head and Neck Surgery, University Medicine Greifswald, Greifswald, Germany; ^6^ Clinical Cancer Research Consulting (CCRC), Düsseldorf, Germany; ^7^ Department of Hematology, Oncology, Hemostaseology and Stem Cell Transplantation, Medical Faculty, RWTH Aachen University, Aachen, Germany; ^8^ Department of Otolaryngology, Head and Neck Surgery, University of Leipzig, Leipzig, Germany; ^9^ University Hospital Würzburg, Bavarian Cancer Research Center (BZKF), Würzburg, Germany; ^10^ Department of Hematology, Oncology, Stem-Cell Transplantation and Palliative Care, Klinikum Stuttgart, Stuttgart, Germany; ^11^ Department of Internal Medicine III (Hematology, Oncology), University Medical Center Mainz, Mainz, Germany; ^12^ Hematology-Oncology Practice Eppendorf (HOPE), Hamburg, Germany

**Keywords:** head and neck squamous cell carcinoma (HNSCC), UV1, pembrolizumab, cancer vaccine, cancer immunotherapy

## Abstract

**Background:**

Globally, head and neck squamous cell carcinoma (HNSCC) is the seventh most common malignancy. Despite aggressive multimodal treatment approaches, recurrent and/or metastatic (R/M) disease develops in >50% of patients. In this setting, pembrolizumab was approved for patients with PD-L1 expression. However, response rates with checkpoint inhibitor monotherapy remain limited and strategies to strengthen tumor-directed immune responses are needed.

**Objective:**

The FOCUS trial is designed to estimate the effectiveness of UV1 vaccination in combination with pembrolizumab versus pembrolizumab as a single agent in patients with R/M HNSCC.

**Methods and analysis:**

The FOCUS trial is a two-armed, randomized, multicenter phase II study which was designed to evaluate the efficacy and feasibility of the hTERT-targeted cancer vaccine UV1 as add-on to pembrolizumab in the 1st line treatment of patients with R/M PD-L1 positive (combined positive score ≥1) HNSCC. Secondary objectives are the exploration of patient subgroups most likely deriving benefit from this novel combination and the establishment of liquid biopsy tumor monitoring in HNSCC.

**Ethics and dissemination:**

This clinical study was designed and will be conducted in compliance with Good Clinical Practice and in accordance with the Declaration of Helsinki. It is intended to publish the results of this study in peer-reviewed scientific journals and to present its content at academic conferences.

**Conclusions:**

A significant number of patients with R/M HNSCC are frail and may not tolerate chemotherapy, these patients may only be suitable for pembrolizumab monotherapy. However, long term disease stabilizations remain the exception and there is a need for the development of efficacious combination regimens for this patient population. The FOCUS study aims to optimize treatment of R/M HNSCC patients with this promising new treatment approach.

**Clinical Trial Registration:**

https://clinicaltrials.gov/study/NCT05075122, identifier NCT05075122.

## 1 Introduction

Worldwide, head and neck squamous cell carcinoma (HNSCC) is the seventh most common malignancy with more than 660,000 new cases and 350,000 deaths per year (1). Risk factors include tobacco use, alcohol consumption, and human papilloma virus (HPV) infection (2).

At early stages, therapy is given with curative intent. However, despite aggressive treatment with multimodal approaches, recurrent and/or metastatic (R/M) disease develops in more than half of patients with HNSCC and prognosis of these patients is poor (3). Many patients suffering from R/M disease present with unresectable disease and only qualify for palliative treatment (4). Until 2019, the EXTREME regimen (cetuximab combined with platinum and fluorouracil) was the standard of care first line treatment for patients with R/M HNSCC with good performance status (ECOG 0-1) (5). More recently, pembrolizumab was approved for R/M HNSCC as monotherapy or in combination with platinum-fluorouracil for PD-L1 positive disease. Approval was based on the KEYNOTE-048 trial, a randomized, phase 3 study, which showed a significant survival benefit when compared with the EXTREME protocol (6). In this trial, pembrolizumab plus chemotherapy improved overall survival compared to cetuximab plus chemotherapy (median 13.0 *vs*. 10.7 months, HR 0.77 [95% CI 0.63-0.93], p=0.0034). In the subgroups of patients with PD-L1 CPS ≥1 and CPS of ≥20, pembrolizumab given as a single agent improved overall survival compared to cetuximab plus chemotherapy (12.3 *vs*. 10.3 months, HR 0.78 [95% CI 0.64-0.96], p=0.0086, and 14.9 *vs*. 10.7 months, HR 0.61 [95% CI 0.45-0.83], p=0.0007) demonstrating increased efficacy of pembrolizumab with increasing PD-L1 expression (7).

Although some patients have durable responses to immune-checkpoint inhibitors, many patients with R/M HNSCC either show no response or benefit only in the short-term from this treatment (3). One reason might be an insufficient T cell effector response (8). To improve the T cell response against tumor antigens, therapeutic cancer vaccines in combination with immune-checkpoint inhibitors are investigated in HNSCC and other tumor entities (8, 9). UV1 is a peptide vaccine targeting human telomerase reverse transcriptase (hTERT), found to be activated in 85-90% of all cancers (8) representing an essential step in carcinogenesis (10). The UV1 vaccine induced persistent immune responses which lasted up to 7.5 years in phase I clinical trials which included patients with non-small cell lung cancer, malignant melanoma, and prostate cancer (8). When combined with the checkpoint-inhibitor ipilimumab, the vaccine-induced T cell response in the melanoma trial occurred more often and more rapidly indicating improved efficacy with the combined approach (8). In patients with advanced melanoma, UV1 was also combined with pembrolizumab (11). In this phase I clinical trial, treatment was well tolerated and response rate was 60% with a 1-year survival rate of 85% (11).

In patients with HNSCC, the combination of immune-checkpoint inhibition with UV1 has not been studied. In 75-100% of HNSCC high levels of hTERT expression have been detected (12). The most common mechanism of hTERT activation are mutations in the promoter region of hTERT (13). In HNSCC frequencies of hTERT promoter mutations vary among different studies (14). Frequencies up to 64,7% have been reported depending on tumor site, risk factors such as human papillomavirus status and ethnicity (14). Thus, hTERT represents an attractive target for therapeutic vaccination in HNSCC.

The FOCUS trial was designed to estimate the effectiveness of UV1 vaccination in combination with pembrolizumab versus pembrolizumab as a single agent in patients with R/M HNSCC.

## 2 Methods and analysis

### 2.1 Study objective

The primary objective of this study is to assess the efficacy of UV1 vaccination in combination with pembrolizumab in patients with R/M HNSCC and PD-L1 CPS ≥1 based on progression free survival according to iRECIST (progression-free survival rate at 6 months after randomization, PFS@6) (15).

Secondary clinical endpoints of this study are overall survival, objective response rate and duration of response according to iRECIST. Other secondary objectives are the UV1 vaccine induced immune responses and the clearance rate of ctDNA from blood during treatment. Additionally, this study will explore the safety and tolerability of UV1 vaccination in combination with pembrolizumab according to NCI CTCAE v5.0. Other objectives are the exploration of what patient subgroups benefit most from this combined approach and the establishment of liquid biopsy tumor monitoring in HNSCC.

### 2.2 Study design

**Figure 1:**
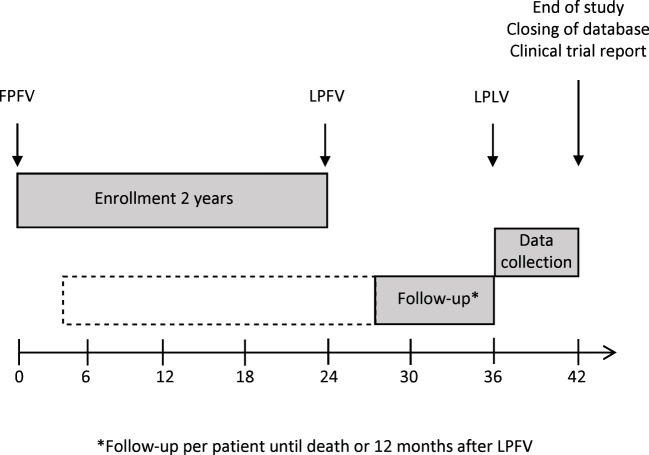
Study recruitment and follow-up. FPFV, first patient, first visit; LPFV, last patient, first visit; LPLV, last patient, last visit.

The FOCUS trial is an open-label, randomized, phase II study which investigates the tolerability and efficacy of the UV1 vaccine in patients with R/M PD-L1 positive (CPS ≥1) HNSCC planned for first-line treatment with pembrolizumab. The study is multicentric and includes several study sites in Germany. 75 patients will be randomized with an estimated recruitment phase of 24 months. Planned duration of follow-up per patient is until death or 12 months after last patient in (**Figure 1**).

### 2.3 Treatment

**Figure 2:**
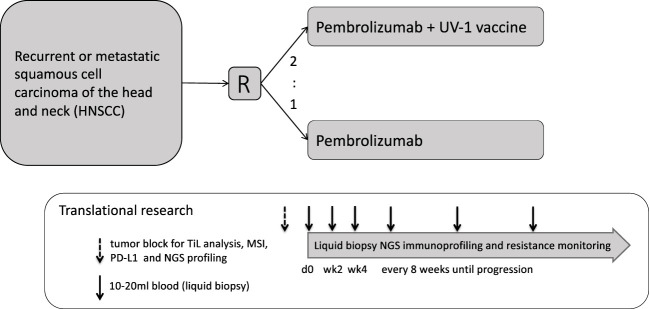
Study schedule.

Eligible patients (**Table 1**) will be randomized to either pembrolizumab, Arm A, about 25 patients, or pembrolizumab in combination with UV1 vaccination plus sargramostim (GM-CSF) as an adjuvant, Arm B, about 50 patients (**Figure 2**).

**Table 1:** 

Inclusion criteria
• Males and Females who are at least 18 years of age • Histologically confirmed diagnosis of a non-resectable recurrent or metastatic head and neck squamous cell carcinoma (not necessarily reconfirmed at time of enrolment) • At least one measurable tumor lesion as per RECIST v1.1, (scan not older than 4 weeks before randomization) • Eligible for pembrolizumab monotherapy (PD-L1 CPS ≥1 and adequate laboratory parameters for pembrolizumab monotherapy as assessed by the investigator) • ECOG-performance score 0-2 • Written informed consent obtained according to international guidelines and local laws • Ability to understand and give informed consent • Safe contraception measures for males and females. Procedures with a pearl index of less than 1% apply as safe pregnancy prevention measures

All patients will receive pembrolizumab until disease progression and up to two years in both arms.

The UV1 vaccine (Ultimovacs, Oslo, Norway) and sargramostim are considered investigational medical products (IMPs) in this study.

Data on efficacy in in terms of vaccine-specific immune response and safety from completed phase I/II clinical trials support a total of 8 vaccinations with a UV1 dose of 300µg administered intradermally with 75µg of the adjuvant sargramostim (8). The administration regimen for UV1 vaccination during day 1-10 is optimized for effective priming and expansion of naïve hTERT-specific T cells in the local lymph nodes draining the vaccine injection site. The following vaccinations are optimized for re-activation of T cell effector activity in the tumor microenvironment in synergy with pembrolizumab.

*Arm A:*

Patients in arm A receive pembrolizumab at 200mg flat dose iv every 3 weeks. Administration starts at week 1 (one week earlier than arm B). The duration of treatment will be 12 weeks.

*Arm B:*

Patients in arm B receive pembrolizumab at 200mg iv every 3 weeks in combination with UV1 vaccination (300µg UV1 plus 75µg GM-CSF as adjuvant). Three UV1 vaccinations are applied during the week before initiation of pembrolizumab, followed by 5 vaccinations applied every 3 weeks on d1 of each cycle (5 cycles in total). Administration of pembrolizumab starts at week 2. In total, the duration of treatment will be 13 weeks.

### 2.4 Assessments

Baseline assessment is performed according to **Table 2**. Radiological imaging by computed tomography (CT) of the neck, chest, abdomen and pelvis according to RECIST v1.1 should not be older than 4 weeks before randomization.

**Table 2:** 

Baseline assessment
• Informed consent • Review of inclusion and exclusion criteria • Relevant medical history • Laboratory tests: Hematology panel, chemistry panel, including also TSH, fT3/fT4, PT/PTT, INR/Quick • Hepatitis B/C screening test, HIV screening test (not older than 4 weeks before randomization) • Physical examination • Vital signs and ECOG • Radiological imaging by computertomography of the neck, chest, abdomen and pelvis (according to RECIST v1.1, not older than 4 weeks before randomization) • Urine pregnancy test • Concomitant medication • C-lab: Stored tumor tissue collection (paraffin block or 10 slides), remaining tumor tissue from the first diagnosis or at relapse • C-lab: Fecal sample

Assessments during treatment will be done on visit 1 (week 1 [W1] day 1 [D1]), visit 2 [W1 D3], visit 3 [W1 D5], visit 4 [W2], visit 5 [W5], visit 6 [W8], visit 7 [W11], visit 8 [W14] and end of treatment (EOT) according to **Table 3**. Screening and visit 1 can be performed on the same day. Assessments at progressive disease (PD) during treatment (if applicable) will be performed according to **Table 4**.

**Table 3:** 

Assessments during treatment
• Vital signs and ECOG (only for visit 1, 4, 5, 6, 7, 8 and EOT) • Laboratory tests: Hematology panel, chemistry panel (only for visits 1, 4, 5, 6, 7 and 8) • TSH, fT3/fT4 at visits 4, 5, 6, 7, 8 and EOT • Urine pregnancy test at visits 5, 6 and 7 • C-lab blood sampling (20ml) for tumor-DNA (only at visits 1, 5, 6, 8 and EOT) • On site preparation and storage: blood sampling (50ml) for PBMC, arm B only (for both arms at site 01 Halle) at visits 1 and 6 • Pembolizumab infusion (arm A) every 3 weeks according to the label at Visits 4, 5, 6, 7 and EOT (Visit 8) • Additional UV1 vaccination (arm B) 3 times the week before initiation of pembrolizumab followed by 5 additional applications on d1 cycle 1-5. • Adverse events • Concomitant medication • Radiological response (according to iRECIST) will be assessed at visit 8 (routine diagnostics)

**Table 4:** 

Assessments at progressive disease (PD) during treatment
• Vital signs and ECOG • Laboratory tests: Hematology panel, chemistry panel • TSH, fT3/fT4 • C-lab blood sampling (20ml) for tumor-DNA • On site preparation and storage: blood sampling (50ml) for PBMC, arm B only (for both arms at site 01 Halle) • Adverse events • Concomitant medication • Radiological response (according to iRECIST) (routine diagnostics) until PD • If not done at screening: C-lab: Stored tumor tissue collection (paraffin block or 10 slides)

### 2.5 Follow-up

All patients will be evaluated every 3 months after EOT until death or maximal 12 months after last patient in (**Table 5**). At progressive disease during follow-up (if not progressed during treatment), assessments will be done according to **Table 6**. All patients will be monitored 30 days after EOT for safety reasons (**Table 7**).

**Table 5:** 

Follow-up
• Results of Radiological imaging (according to iRECIST) regarding disease status (from routine diagnostics) until PD • Pembrolizumab infusion is continued according to SmPC at the discretion of the physician • C-lab blood sampling (20ml) for tumor-DNA (only at FU1 and FU2) • Assessment of adverse events, concomitant therapies (only until FU1), subsequent anti-cancer therapies and survival status

**Table 6:** 

Visit at PD during follow up (if not progressed during treatment)
• Results of Radiological imaging (according to iRECIST) regarding disease status (from routine diagnostics) • Subsequent anti-cancer therapies • C-lab blood sampling (20ml) for tumor-DNA • Survival and disease status • Assessment of adverse events, concomitant therapies and subsequent anti-cancer therapies • Blood sampling (50ml) for PBMC (on site preparation and storage) arm B only (for both arms at site 01 Halle) • If not done at screening: C-lab: Stored tumor tissue collection (paraffin block or 10 slides)

**Table 7:** 

Safety follow-up
• Vital signs and ECOG status • Physical examination • Laboratory tests: Hematology panel, chemistry panel, including also TSH, fT3/fT4 • C-lab blood sampling (20ml) for tumor-DNA • Pembrolizumab infusion is continued according to SmPC at the discretion of the physician • Assessment of adverse events, concomitant therapies and subsequent anti-cancer therapies

### 2.6 Sample collection for biomarker program

Blood samples for immune analysis (**Table 8**) will be collected at visit 1, 5, 6, 8 (EOT) as well as at safety follow-up (FU), FU1, FU2 and at PD (**Figure 2**).

**Table 8:** 

Translational work-up
• Immunohistochemistry (IHC) for PD-L1 to deduce tumor proportion score (TPS), immune cell (IC) score and combined positivity score (CPS), and IHC for telomerase tissue expression • Next-generation T cell receptor repertoire sequencing of circulating and tumor-infiltrating lymphocytes (TiL) • Next-generation gene panel sequencing for mutational profiling of tumor or circulating tumor DNA (ctDNA, liquid biopsy) • In individual patients liquid biopsy courses will be confirmed with digital droplet PCR (ddPCR) as an alternative methodology • Immune response assays against hTERT peptides measured by 3H-Thymidine proliferation and IFNgamma ELISPOT assays

In centers with expertise in collecting and locally freezing peripheral blood mononuclear cells (PBMCs), additional blood will be collected at visit 1, 6 and at PD from patients receiving UV1 vaccination (from patients of both arms only at site 01 Halle only) for immune response assays (**Table 8**).

Tumor tissue acquired before treatment initiation at first diagnosis or at relapse (biopsy of primary tumor, surgical material, or biopsy material of metastatic lesions) and potential biopsy or surgical material acquired during the study will be analyzed (**Table 8**, **Figure 2**).

Fecal samples will be collected prior to treatment to evaluate the gut microbiome (**Table 8**).

### 2.7 Analysis of primary study endpoint

The primary study endpoint progression PFS@6 will be analyzed as the proportion of all intention-to-treat patients being known to be alive without progression at 6 months after randomization, providing the 95%, 90% and 80% confidence intervals for this estimate.

### 2.8 Statistics and data handling

The FOCUS trial is designed as a randomized phase II study to estimate the therapeutic effectiveness of pembrolizumab in combination with UV1 vaccination in relation to the standard treatment (single drug pembrolizumab). The assumptions on outcome after standard therapy is derived from available data and controlled for by a randomized reference (or calibration) arm. Due to this design, analysis of both treatment arms but no formal statistical comparison will be performed. All secondary endpoint analyses are considered explorative. Further clinical development of this combination depends on the primary endpoint (and its confidence interval), the findings in the control arm, and the supporting safety and feasibility findings.

### 2.9 Sample size estimation

The progression-free survival rate after 6 months (PFSR@6) with single agent pembrolizumab as first-line treatment in R/M HNSCC is about 25%. Pembrolizumab in combination with UV1 vaccination should result in a PFSR@6 of 40% to be regarded as promising for further development in a phase III setting. Based on these assumptions and by applying a one-sided test with an alpha error level of 0.1 and a beta error of 0.2 (corresponding to a power of 80%), 46 evaluable patients are needed in the experimental arm. According to the 2:1 randomization, about 23 patients will be included in the control arm. To allow for a 10% drop out rate, a total of 75 patients should be included.

## 3 Discussion

Patients with R/M HNSCC have a poor prognosis (3, 4). Many of these patients are frail and cannot tolerate chemotherapy.

Eligible patients with R/M HNSCC may be treated with immunotherapy. Nivolumab was shown to significantly prolong survival when compared with standard systemic therapy in patients progressing within six months after platinum-chemotherapy (16). Due to these promising results, patients in the KEYNOTE-048 trial (6) received either the EXTREME regimen, or pembrolizumab as a single agent, or platinum/5-fluorouracil with pembrolizumab as first-line therapy (17). After a follow-up of 4 years, a survival benefit and a longer duration of response was observed with single-agent pembrolizumab and pembrolizumab in combination with chemotherapy compared with cetuximab in combination with chemotherapy (18). Two phase III studies evaluated the combination of anti-PD1/PDL1 and anti-CTLA antibodies in R/M HNSCC patients (19, 20). In both studies the combination proved to be tolerable but showed no statistically significant improvement in overall survival versus the EXTREME protocol (CheckMate 651) or single agent standard of care (EAGLE) (19, 20). The combination of checkpoint inhibition plus cetuximab was investigated in two phase II studies in patients with R/M HNSCC (21, 22). Pembrolizumab plus cetuximab had an overall response rate of 45% (21). Nivolumab plus cetuximab was also effective in pretreated patients with a 1-year overall survival of 50% (22). These studies provide a rationale for a larger randomized study. In a phase IB/II trial of the angiogenesis inhibitor lenvatinib plus pembrolizumab which included 22 HNSCC patients, these patients had an objective response rate of 36% at week 24 (23). However, frail patients may not tolerate this regimen and the development of efficacious combination regimens for this patient population is urgently needed.

The FOCUS trial investigates the tolerability and efficacy of the cancer vaccine UV1 combined with first-line pembrolizumab monotherapy in patients with R/M HNSCC and CPS ≥1.

The therapeutic cancer UV1 consists of three synthetic peptides which cover a sequence within the active catalytic site of hTERT (24). hTERT promoter mutations which are a common mechanism of hTERT activation are found in the two major hotspots C228T and C250T (14). In HNSCC hTERT promoter mutations were found to be associated with poorer overall survival in some studies (25, 26).

UV1 vaccination was investigated in phase I trials in patients with metastatic prostate cancer combined with androgen blockade (27) and as monotherapy in patients with stage III/IV non-small cell lung cancer (NSCLC) (24).

Patients with metastatic prostate cancer (n=22) were treated with 3 dose levels of UV1 combined with GM-CSF (27). In this study, treatment with UV1 was well tolerated and specific immune responses were noted in 18 of 21 patients (27).

UV1 treatment was also safe and immunogenic in patients with advanced NSCLC (24). 18 patients with advanced stage NSCLC without brain metastasis were enrolled (24). Patients who did not show an immune response had a median overall survival of 21.3 months whereas the overall survival was 38.4 months in patients who did show an immune response (24).

Long-term monitoring revealed a persistent telomerase peptide-specific immune response which lasted up to 7.5 years following the initial vaccination (8).

Vaccine-based therapies may be more effective in combination with other immunotherapies as the immunosuppressive environment of the tumor may interfere with vaccine-activated T cells (9). By way of example, checkpoint-inhibitors block the immunosuppression induced by the PD-1/PD-L1 axis which may be accompanied by a more efficient vaccine mediated anti-tumor T cell response (9).

Potential synergistic effect of cytotoxic T-lymphocyte-associated protein 4 (CTLA-4) blockade and hTERT vaccination was investigated in metastatic melanoma in a phase I/IIa clinical trial (28). 12 melanoma patients were treated with UV1 in combination with ipilimumab (28). Most patients had T cell responses to UV1 peptides, 3 patients partially responded, one patient had a complete response, and overall survival was 50% at 5 years (28). Adverse events included diarrhea, rash, pruritus, fatigue, and nausea (28). The combination of UV1 and ipilimumab was safe and toxicity was mainly low-grade (28), however, patients in the FOCUS study will be carefully evaluated for potential toxicities. All patients will be monitored 30 days after EOT for safety reasons.

Four phase II studies currently evaluate the combination of different checkpoint inhibitors plus UV1 vaccination in metastatic malignant melanoma (NCT04382664), mesothelioma (NCT04300244), ovarian cancer (NCT04742075), and non-small cell lung cancer (NCT05344209).

To identify potential patients that benefit most from the combination of UV1 and pembrolizumab and to uncover potential mechanisms of resistance, the FOCUS trial is accompanied by a biomarker program which includes assessment of tumor biopsies prior to treatment, immunomonitoring by next-generation sequencing (NGS), and liquid biopsy monitoring of tumor subclones during treatment.

The peripheral blood T cell space shows age-specific architectures with cancer patients overall displaying reduced repertoire richness and diversity (29). Previous studies showed that immune checkpoint blockade led to diversification of the peripheral blood T cell space in patients with melanoma and other solid tumors, which was associated with response to treatment in some studies (30–32). In the FOCUS study, the characteristics of tumor-infiltrating lymphocytes and blood-circulating T cells will be studied by NGS and immunological analyses will be correlated with vaccine-specific immune responses assessed by standardized T cell proliferation assays.

Furthermore, tumor tissue and liquid biopsy testing will be done at baseline using a gene panel which covers frequent driver and resistance mutations in HNSCC. The circulating tumor DNA clearance over time will be correlated with overall response, progression-free survival, and overall survival. To search for tumor subclones potentially resistant to pembrolizumab or UV1, the liquid biopsy panel includes genes involved in resistance to checkpoint inhibitors as well as the coding region of hTERT as the UV1 target.

In conclusion, the FOCUS trial investigates the potential synergistic effect of UV1 vaccination and checkpoint blockade with pembrolizumab in patients with R/M HNSCC. To optimize tumor response in this often frail and pretreated patient population, an extensive biomarker program accompanies the FOCUS trial.

## Data availability statement

The original contributions presented in the study are included in the article/supplementary material. Further inquiries can be directed to the corresponding author.

## Author contributions

AB: Writing – original draft, Writing – review & editing. CS: Conceptualization, Writing – review & editing. KK: Conceptualization, Writing – review & editing. PS: Conceptualization, Writing – review & editing. C-JB: Conceptualization, Writing – review & editing. MBl: Conceptualization, Writing – review & editing. AH: Conceptualization, Writing – review & editing. MT: Conceptualization, Writing – review & editing. AD: Conceptualization, Writing – review & editing. UM-R: Conceptualization, Writing – review & editing. DH: Conceptualization, Writing – review & editing. JA: Conceptualization, Writing – review & editing. AS: Conceptualization, Writing – review & editing. MBi: Conceptualization, Writing – original draft, Writing – review & editing.

## References

[1] Gormley M Creaney G Schache A Ingarfield K Conway DI. Reviewing the epidemiology of head and neck cancer: definitions, trends and risk factors. Br Dent J (2022) 233(9):780–6. doi: 10.1038/s41415-022-5166-x PMC9652141 36369568

[2] Johnson DE Burtness B Leemans CR Lui VWY Bauman JE Grandis JR. Head and neck squamous cell carcinoma. Nat Rev Dis Primers (2020) 6(1):92. doi: 10.1038/s41572-020-00224-3 33243986 PMC7944998

[3] Chow LQM. Head and neck cancer. N Engl J Med (2020) 382(1):60–72. doi: 10.1056/NEJMra1715715 31893516

[4] Lau A Yang WF Li KY Su YX. Systemic therapy in recurrent or metastatic head and neck squamous cell carcinoma- A systematic review and meta-analysis. Crit Rev Oncol Hematol (2020) 153:102984. doi: 10.1016/j.critrevonc.2020.102984 32569853

[5] Vermorken JB Mesia R Rivera F Remenar E Kawecki A Rottey S. Platinum-based chemotherapy plus cetuximab in head and neck cancer. N Engl J Med (2008) 359(11):1116–27. doi: 10.1056/NEJMoa0802656 18784101

[6] Burtness B Harrington KJ Greil R Soulières D Tahara M de Castro G. Pembrolizumab alone or with chemotherapy versus cetuximab with chemotherapy for recurrent or metastatic squamous cell carcinoma of the head and neck (KEYNOTE-048): a randomised, open-label, phase 3 study. Lancet (2019) 394(10212):1915–28. doi: 10.1016/S0140-6736(19)32591-7 31679945

[7] Burtness B Rischin D Greil R Soulières D Tahara M de Castro G. Pembrolizumab alone or with chemotherapy for recurrent/metastatic head and neck squamous cell carcinoma in KEYNOTE-048: subgroup analysis by programmed death ligand-1 combined positive score. J Clin Oncol (2022) 40(21):2321–32. doi: 10.1200/JCO.21.02198 PMC9287281 35333599

[8] Ellingsen EB Aamdal E Guren T Lilleby W Brunsvig PF Mangsbo SM. Durable and dynamic hTERT immune responses following vaccination with the long-peptide cancer vaccine UV1: long-term follow-up of three phase I clinical trials. J Immunother Cancer (2022) 10(5). doi: 10.1136/jitc-2021-004345 PMC9134181 35613827

[9] Beyaert S Machiels JP Schmitz S. Vaccine-based immunotherapy for head and neck cancers. Cancers (Basel) (2021) 13(23). doi: 10.3390/cancers13236041 PMC8656843 34885150

[10] Hanahan D Weinberg RA. Hallmarks of cancer: the next generation. Cell (2011) 144(5):646–74. doi: 10.1016/j.cell.2011.02.013 21376230

[11] Ellingsen EB O’Day S Mezheyeuski A Gromadka A Clancy T Kristedja TS. Clinical activity of combined telomerase vaccination and pembrolizumab in advanced melanoma: results from a phase I trial. Clin Cancer Res (2023) 29:3026–36. doi: 10.1158/1078-0432.c.6736744.v2 PMC10425723 37378632

[12] Boscolo-Rizzo P Da Mosto MC Rampazzo E Giunco S Del Mistro A Menegaldo A. Telomeres and telomerase in head and neck squamous cell carcinoma: from pathogenesis to clinical implications. Cancer Metastasis Rev (2016) 35(3):457–74. doi: 10.1007/s10555-016-9633-1 PMC5035656 27501725

[13] Ellingsen EB Mangsbo SM Hovig E Gaudernack G. Telomerase as a target for therapeutic cancer vaccines and considerations for optimizing their clinical potential. Front Immunol (2021) 12:682492. doi: 10.3389/fimmu.2021.682492 34290704 PMC8288190

[14] Yeh TJ Luo CW Du JS Huang CT Wang MH Chuang TM. Deciphering the functions of telomerase reverse transcriptase in head and neck cancer. Biomedicines (2023) 11(3). doi: 10.3390/biomedicines11030691 PMC10044978 36979671

[15] Seymour L Bogaerts J Perrone A Ford R Schwartz LH Mandrekar S. iRECIST: guidelines for response criteria for use in trials testing immunotherapeutics. Lancet Oncol (2017) 18(3):e143–e52. doi: 10.1016/S1470-2045(17)30074-8 PMC5648544 28271869

[16] Ferris RL Blumenschein G Jr. Fayette J Guigay J Colevas AD Licitra L. Nivolumab for recurrent squamous-cell carcinoma of the head and neck. N Engl J Med (2016) 375(19):1856–67. doi: 10.1056/NEJMoa1602252 PMC5564292 27718784

[17] Szturz P Vermorken JB. Translating KEYNOTE-048 into practice recommendations for head and neck cancer. Ann Transl Med (2020) 8:975. doi: 10.21037/atm.2020.03.164 32953775 PMC7475419

[18] Harrington KJ Burtness B Greil R Soulières D Tahara M de Castro G Jr.. Pembrolizumab with or without chemotherapy in recurrent or metastatic head and neck squamous cell carcinoma: updated results of the phase III KEYNOTE-048 study. J Clin Oncol (2023) 41(4):790–802. doi: 10.1200/JCO.21.02508 36219809 PMC9902012

[19] Haddad RI Harrington K Tahara M Ferris RL Gillison M Fayette J. Nivolumab plus ipilimumab versus EXTREME regimen as first-line treatment for recurrent/metastatic squamous cell carcinoma of the head and neck: the final results of checkMate 651. J Clin Oncol (2023) 41(12):2166–80. doi: 10.1200/JCO.22.00332 PMC10115555 36473143

[20] Ferris RL Haddad R Even C Tahara M Dvorkin M Ciuleanu TE. Durvalumab with or without tremelimumab in patients with recurrent or metastatic head and neck squamous cell carcinoma: EAGLE, a randomized, open-label phase III study. Ann Oncol (2020) 31(7):942–50. doi: 10.1016/j.annonc.2020.04.001 32294530

[21] Sacco AG Chen R Worden FP Wong DJL Adkins D Swiecicki P. Pembrolizumab plus cetuximab in patients with recurrent or metastatic head and neck squamous cell carcinoma: an open-label, multi-arm, non-randomised, multicentre, phase 2 trial. Lancet Oncol (2021) 22(6):883–92. doi: 10.1016/S1470-2045(21)00136-4 PMC12140401 33989559

[22] Chung CH Li J Steuer CE Bhateja P Johnson M Masannat J. Phase II multi-institutional clinical trial result of concurrent cetuximab and nivolumab in recurrent and/or metastatic head and neck squamous cell carcinoma. Clin Cancer Res (2022) 28(11):2329–38. doi: 10.1158/1078-0432.CCR-21-3849 PMC9167762 35344035

[23] Taylor MH Lee CH Makker V Rasco D Dutcus CE Wu J. Phase IB/II trial of lenvatinib plus pembrolizumab in patients with advanced renal cell carcinoma, endometrial cancer, and other selected advanced solid tumors. J Clin Oncol (2020) 38(11):1154–63. doi: 10.1200/JCO.19.01598 PMC7145588 31961766

[24] Brunsvig PF Guren TK Nyakas M Steinfeldt-Reisse CH Rasch W Kyte JA. Long-term outcomes of a phase I study with UV1, a second generation telomerase based vaccine, in patients with advanced non-small cell lung cancer. Front Immunol (2020) 11:572172. doi: 10.3389/fimmu.2020.572172 33324397 PMC7726017

[25] Qu Y Dang S Wu K Shao Y Yang Q Ji M. TERT promoter mutations predict worse survival in laryngeal cancer patients. Int J Cancer (2014) 135(4):1008–10. doi: 10.1002/ijc.28728 24436132

[26] Arantes L Cruvinel-Carloni A de Carvalho AC Sorroche BP Carvalho AL Scapulatempo-Neto C. TERT promoter mutation C228T increases risk for tumor recurrence and death in head and neck cancer patients. Front Oncol (2020) 10:1275. doi: 10.3389/fonc.2020.01275 32850388 PMC7399085

[27] Lilleby W Gaudernack G Brunsvig PF Vlatkovic L Schulz M Mills K. Phase I/IIa clinical trial of a novel hTERT peptide vaccine in men with metastatic hormone-naive prostate cancer. Cancer Immunol Immunother (2017) 66(7):891–901. doi: 10.1007/s00262-017-1994-y 28391357 PMC11028648

[28] Aamdal E Inderberg EM Ellingsen EB Rasch W Brunsvig PF Aamdal S. Combining a universal telomerase based cancer vaccine with ipilimumab in patients with metastatic melanoma - five-year follow up of a phase I/IIa trial. Front Immunol (2021) 12:663865. doi: 10.3389/fimmu.2021.663865 34046035 PMC8147687

[29] Simnica D Akyüz N Schliffke S Mohme M VW L Mährle T. T cell receptor next-generation sequencing reveals cancer-associated repertoire metrics and reconstitution after chemotherapy in patients with hematological and solid tumors. Oncoimmunology (2019) 8(11):e1644110. doi: 10.1080/2162402X.2019.1644110 31646093 PMC6791461

[30] Kvistborg P Philips D Kelderman S Hageman L Ottensmeier C Joseph-Pietras D. Anti-CTLA-4 therapy broadens the melanoma-reactive CD8+ T cell response. Sci Transl Med (2014) 6(254):254ra128. doi: 10.1126/scitranslmed.3008918 25232180

[31] Robert L Tsoi J Wang X Emerson R Homet B Chodon T. CTLA4 blockade broadens the peripheral T-cell receptor repertoire. Clin Cancer Res (2014) 20(9):2424–32. doi: 10.1158/1078-0432.CCR-13-2648 PMC4008652 24583799

[32] Akyüz N Brandt A Stein A Schliffke S Mährle T Quidde J. T-cell diversification reflects antigen selection in the blood of patients on immune checkpoint inhibition and may be exploited as liquid biopsy biomarker. Int J Cancer (2017) 140(11):2535–44. doi: 10.1002/ijc.30549 27925177

